# Supplementary material for: Topology of vibrational modes predicts plastic events in glasses
Source: Nat Commun. 2023 May 24;14:2955. doi: 10.1038/s41467-023-38547-w (PMC10209080; doi:10.1038/s41467-023-38547-w)
Supplement: Supplementary file 1 — Supplementary Information [file 41467_2023_38547_MOESM1_ESM.pdf]

Topology of vibrational modes predicts plastic events in glasses

Zhen Wei Wu[1,2], Yixiao Chen[3], Wei-Hua Wang[4] Walter Kob[5], and Limei Xu[2]

[1] Institute of Nonequilibrium Systems, School of Systems Science, Beijing Normal University, 100875 Beijing, China

[2] International Center for Quantum Materials, School of Physics, Peking University, Beijing 100871, China

[3] Yuanpei College, Peking University, 100871 Beijing, China

[4] Institute of Physics, Chinese Academy of Sciences, Beijing 100190, China

[5] Department of Physics, University of Montpellier and CNRS, 34095 Montpellier, France

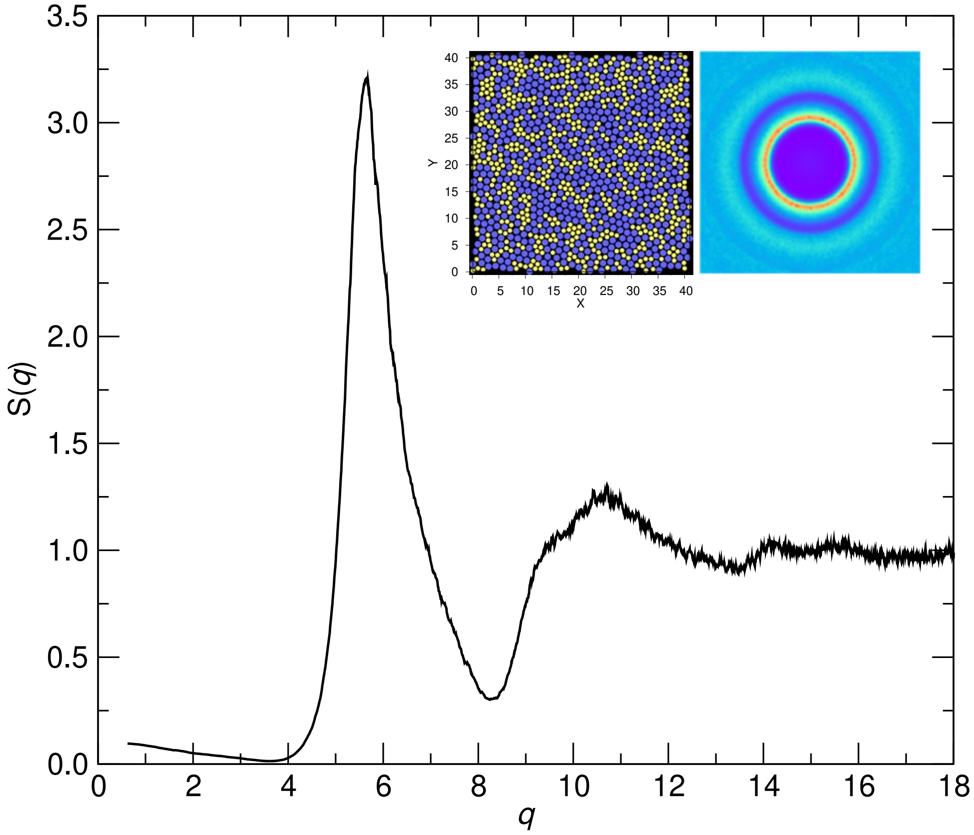

Supplementary Fig. 1. **The static structure factor** $S(q)$ **of the model glass (**N**=1250) at a number density of 0.75 at** T**=0.1.** $S(q)$, calculated from the position of the particles via $S(q) = (1/N)\langle|\sum_j e^{-\mathbf{iq}\cdot\mathbf{r}_j}|^2\rangle$, shows the typical form found in disordered systems. The left inset shows a snapshot of the system and the right inset shows the two-dimensional static structure factor.

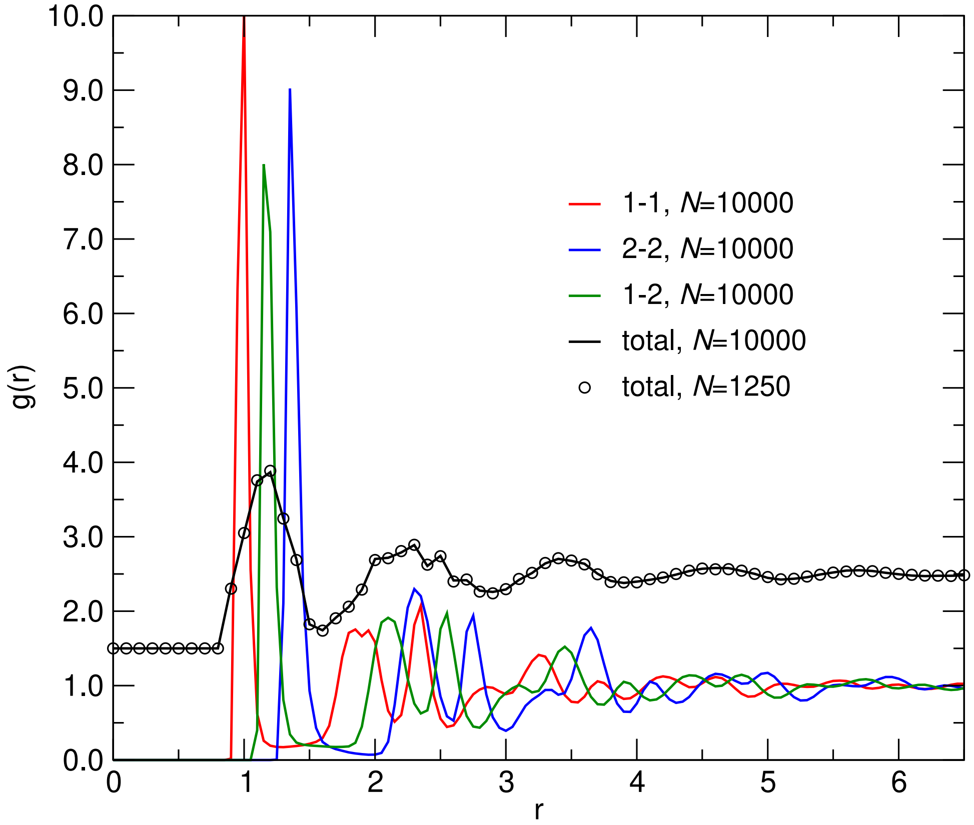

Supplementary Fig. 2. **Radial distribution function.** The colored curves are the partial radial distribution functions as obtained for the system with $N = 10^4$ particles. The resulting total radial distribution function is show in black (shifted vertically by 1.5 for the sake of visibility). The back symbols are the total radial distribution function for the system with $N = 1250$ particles. It superposes perfectly with the data for the larger system, showing that there are no finite size effects.

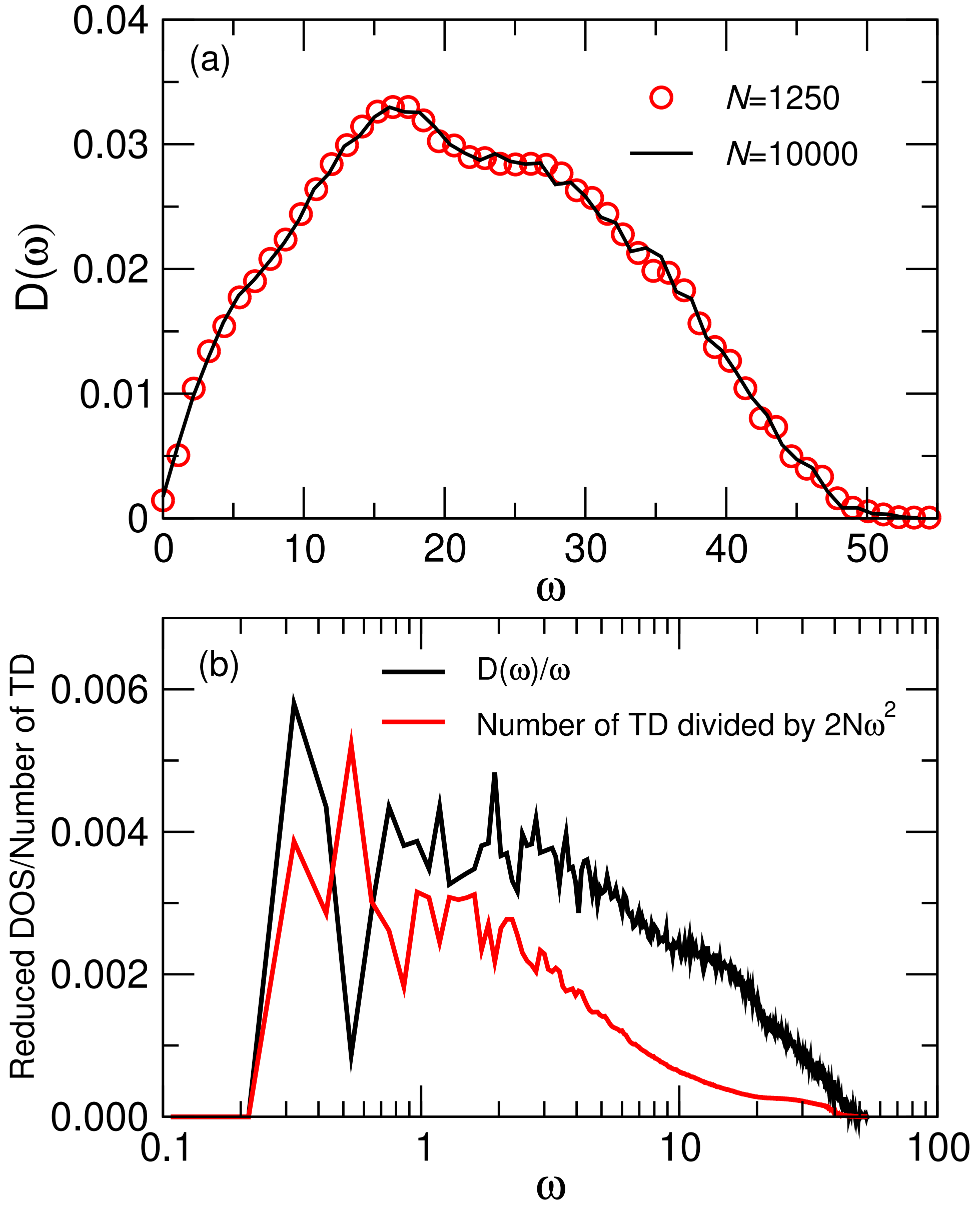

Supplementary Fig. 3. **Vibrational density of states.** **(a)**: Vibrational density of states for two system sizes. No system size dependence is seen. For N=1250, the data has been averaged over 12 independent samples. **(b)**: Vibrational density of states divided by the frequency. At low frequencies this ratio is a constant, thus showing that the Debye law holds at low ω while it breaks down for frequencies above $\omega \approx 3.0$. Also included is the total number of topological defects, divided by $2N\omega^2$, showing that this ratio also starts to decrease at around $\omega = 3.0$.

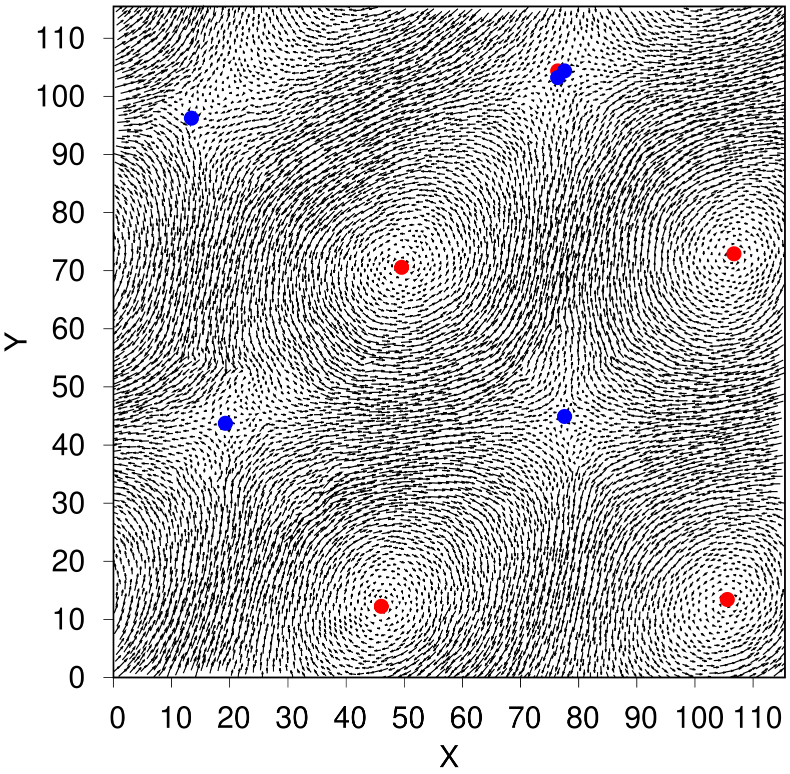

Supplementary Fig. 4. **Ordering of the eigen-vector field and topological defects at low frequencies.** N=10000 and ω=0.51. At small frequencies the vibrational modes are of acoustic nature and hence form a regular pattern, the geometry of which depends on the eigen-mode considered. As a consequence the resulting topological defects show also an ordered arrangement (+1 TDs in red and −1 TDs in blue).

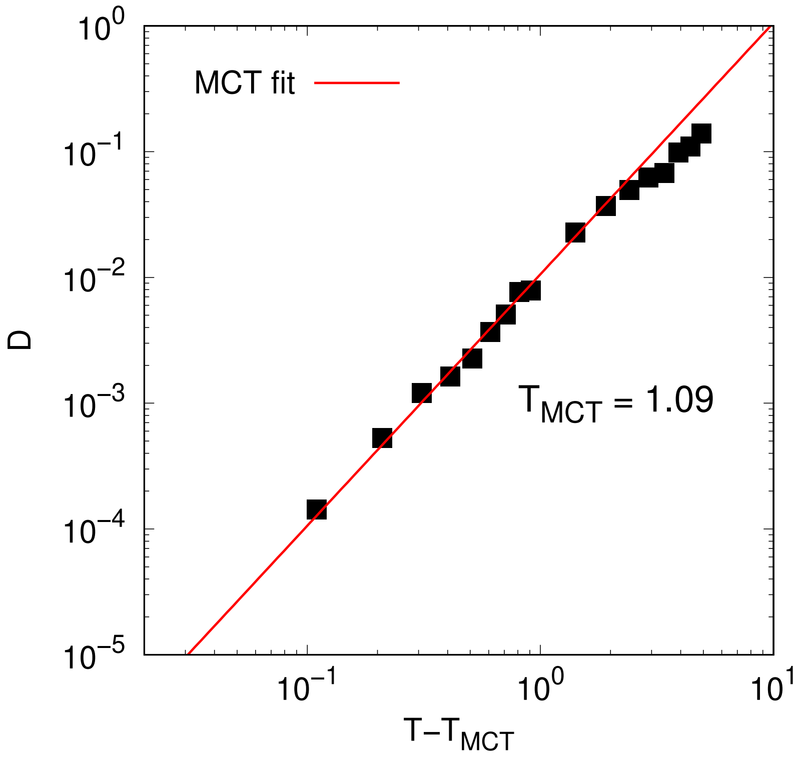

Supplementary Fig. 5. **Temperature dependence of the diffusion constant** D**.** The diffusion constant D has been obtained from the mean squared displacement of the particles and the Einstein relation. To determine D we quenched the system to the target temperature, equilibrated it for 10^6 steps and then made a production run of length 2×10^6 steps. Also included in the graph is a fit with a power-law suggested by mode-coupling theory (MCT) of the form $D \propto (T - T_{\rm MCT})^\gamma$, where $T_{\rm MCT}$ is the critical temperature of the theory. The so obtained values for $T_{\rm MCT}$ and γ are $T_{\rm MCT} = 1.09$ and $\gamma = 2.0$, respectively. Thus the temperature $T = 0.1$ at which we probe our glass sample is deep in the glass state.

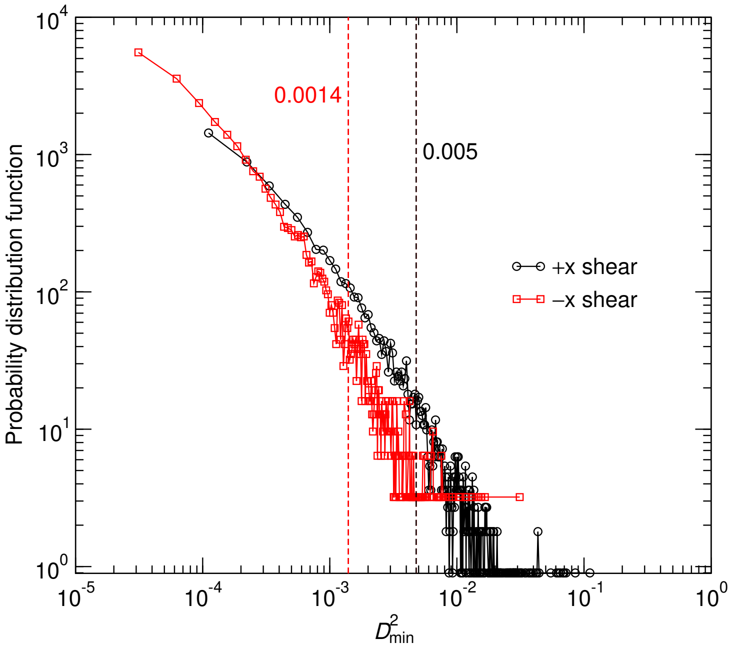

Supplementary Fig. 6. **Probability distribution function of D^2_{min}.** Distribution of the non-affine displacement D^2_{min} after the system has been sheared to the strain 2.5%. The two curves correspond to a shear in the $+x$ and $-x$ directions. Particles that are in the top 5% of the displacement are used to define the plastic events shown in Fig. 3 of the main text. This corresponds to values of $D^2_{\mathrm{min}} > 0.005$ for the $+x$ shear and 0.0014 for the −x shear, respectively, see vertical dashed lines. The difference of these two values are due to finite size effects.

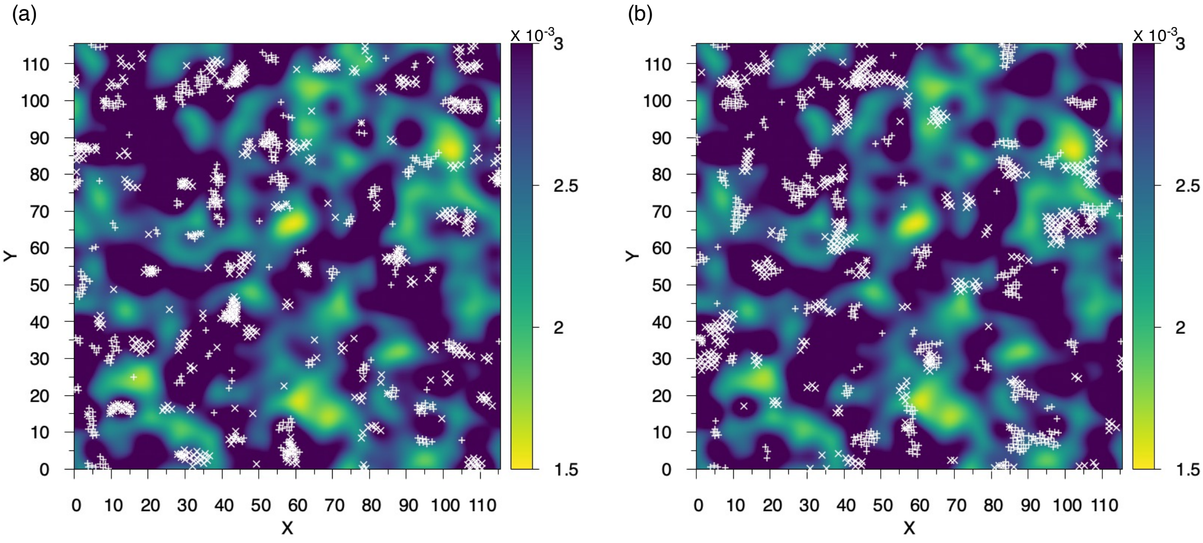

Supplementary Fig. 7. **Number density field of the -1 TDs (color map) and of the PE (symbols) at strain $\gamma = 2.5\%$, (a), and $\gamma = 5\%$, (b).** The density field, in units of TD per unit area, has been obtained by averaging the TDs of all the modes over all frequencies up to $\omega_{\rm max} = 3.5$, using a weight factor ω^{-2}, and a subsequent Gaussian smoothing function of width 4. PE resulting from a shear in the positive and negative x-direction are marked by $+$ and $\times$ filled symbols, respectively. The comparison of the two panels shows that an increase of strain does not modify significantly the pattern of the PE.

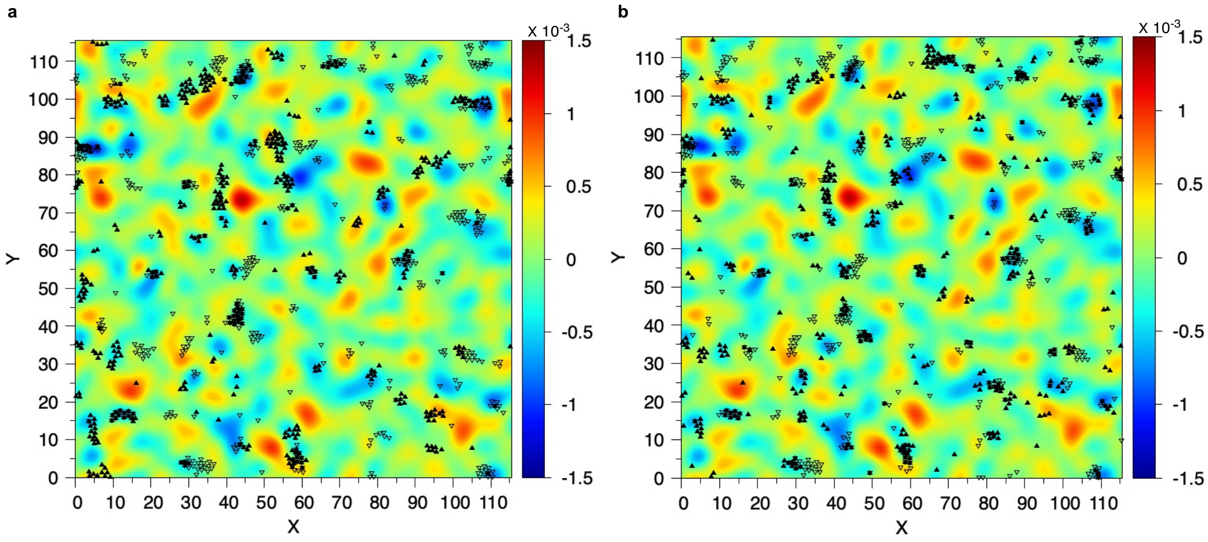

Supplementary Fig. 8. **Charge density field of the TDs (color map) and of the PE's (symbols) at strain $\gamma = 0.025$.** Panel **(a)** shows the PE's resulting from a simple shear parallel to the x-direction and in panel **(b)** the ones for shear parallel to the y-direction. Filled and open symbols correspond to the positive and negative direction, respectively.

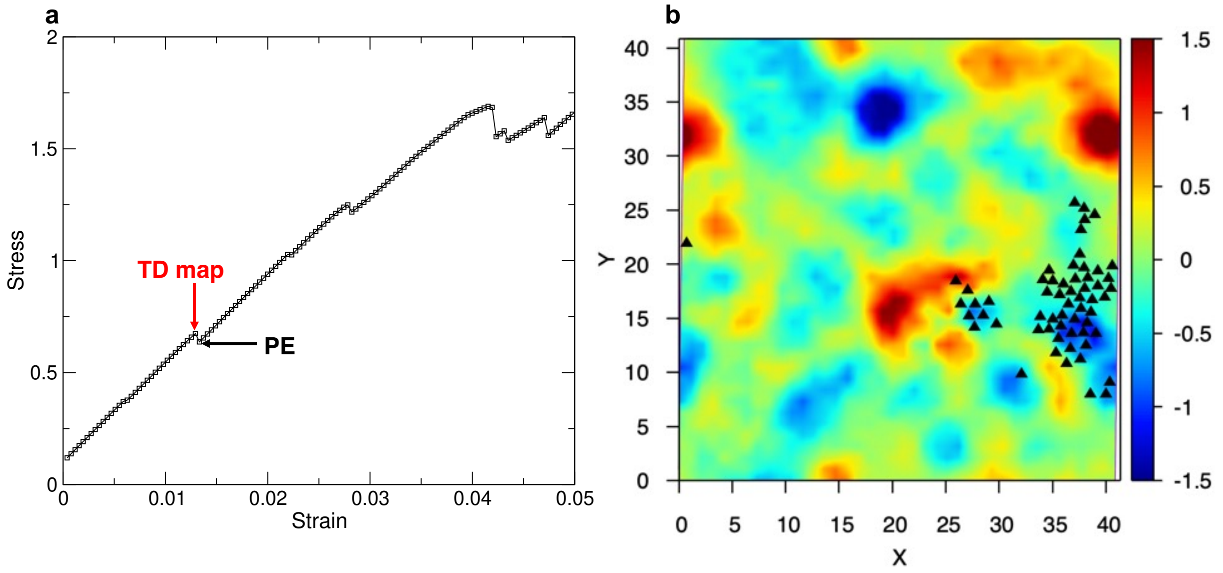

Supplementary Fig. 9. **Stress strain curve and charge density map at strain 0.013. (a)** The stress-strain curve with a mark at the strain at which the charge density map shown in panel **(b)** was constructed. **(b)** The charge density map and the associated PE.
